# 2-Amino­pyridinium 2-meth­oxy­carbonyl-4,6-dinitro­phenolate

**DOI:** 10.1107/S160053681105286X

**Published:** 2011-12-14

**Authors:** Dong-Liang Wu, Zi-Jing Xiao

**Affiliations:** a College of Materials Science & Engineering, Huaqiao University, Xiamen 361021, People’s Republic of China

## Abstract

In the title mol­ecular salt, C_5_H_7_N_2_^+^·C_8_H_5_N_2_O_7_^−^, the 2-amino­pyridinium cation is essentially planar, with a maximium deviation of 0.015 (1) Å, while the 2-meth­oxy­carbonyl-4,6-dinitro­phenolate anion is slightly twisted away from planarity, with a maximium deviation of 0.187 (1) Å. Deprotonation of the hy­droxy O atom was observed. The cation and anion are connected by four bifurcated N—H⋯(O,O) hydrogen bonds, forming a mol­ecular proton-transfer adduct. The dihedral angle between the pyridinium ring in the cation and the benzene ring in the anion is 3.65 (6)°. Every adduct connects to six neighboring adducts by N—H⋯O and C—H⋯O hydrogen bonds, yielding extended layers parallel to the *bc* plane. There is a weak π–π inter­action between the benzene rings of two neighboring anions; the inter­planar spacing and the centroid–centroid separation are 3.309 (1) and 3.69 (1) Å, respectively.

## Related literature

For the structures of mol­ecular proton-transfer adducts containing substituted pyridinium and an acid anion, see Gellert & Hsu (1988 ▶); Smith *et al.* (2000 ▶); Jebas *et al.* (2006 ▶); Rademeyer (2007 ▶); Hemamalini & Fun (2010*a* ▶,*b* ▶,*c* ▶); Perpétuo & Janczak (2010 ▶). For comparable structures, see: Jebas *et al.* (2006 ▶); Perpétuo & Janczak (2010 ▶); Hemamalini & Fun (2010*a* ▶). For the synthesis of 3,5-dinitro­methyl salicylate, see: Bartlett & Trachten (1958 ▶).
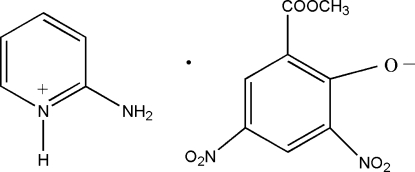


## Experimental

### 

#### Crystal data

C_5_H_7_N_2_^+^·C_8_H_5_N_2_O_7_^−^
*M* *_r_* = 336.27
Monoclinic, 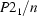

*a* = 7.4088 (3) Å
*b* = 19.1779 (6) Å
*c* = 9.9784 (4) Å
β = 98.2825 (15)°
*V* = 1403.00 (9) Å^3^
*Z* = 4
Mo *K*α radiation
μ = 0.13 mm^−1^
*T* = 293 K
0.35 × 0.35 × 0.26 mm

#### Data collection

Rigaku R-AXIS SPIDER IP diffractometer
Absorption correction: ψ scan (*TEXRAY*; Molecular Structure Corporation, 1999) ▶ *T*_min_ = 0.951, *T*_max_ = 0.969
21789 measured reflections
3200 independent reflections
2760 reflections with *I* > 2σ(*I*)
*R*_int_ = 0.024

#### Refinement

*R*[*F* ^2^ > 2σ(*F* ^2^)] = 0.040
*wR*(*F* ^2^) = 0.111
*S* = 1.09
3200 reflections
221 parameters
H-atom parameters constrained
Δρ_max_ = 0.32 e Å^−3^
Δρ_min_ = −0.30 e Å^−3^

### 

Data collection: *RAPID-AUTO* (Rigaku, 2008 ▶); cell refinement: *RAPID-AUTO*; data reduction: *RAPID-AUTO*; program(s) used to solve structure: *SHELXS97* (Sheldrick, 2008 ▶); program(s) used to refine structure: *SHELXL97* (Sheldrick, 2008 ▶); molecular graphics: *ORTEX* (McArdle, 1995 ▶); software used to prepare material for publication: *SHELXL97*.

## Supplementary Material

Crystal structure: contains datablock(s) I, global. DOI: 10.1107/S160053681105286X/ez2268sup1.cif

Structure factors: contains datablock(s) I. DOI: 10.1107/S160053681105286X/ez2268Isup2.hkl

Supplementary material file. DOI: 10.1107/S160053681105286X/ez2268Isup3.cml

Additional supplementary materials:  crystallographic information; 3D view; checkCIF report

## Figures and Tables

**Table 1:** Hydrogen-bond geometry (Å, °)

*D*—H⋯*A*	*D*—H	H⋯*A*	*D*⋯*A*	*D*—H⋯*A*
N1—H01*A*⋯O3	0.90	1.88	2.6864 (13)	148
N1—H01*A*⋯O2	0.90	2.23	2.8783 (14)	130
N2—H02*A*⋯O3	0.88	2.01	2.7592 (14)	142
N2—H02*A*⋯O4	0.88	2.45	3.2082 (15)	144
N2—H02*B*⋯O6^i^	0.87	2.24	3.0537 (14)	155
C4—H4*A*⋯O7^ii^	0.93	2.57	3.2052 (16)	126
C5—H5*A*⋯O5^iii^	0.93	2.42	3.2604 (17)	151

Symmetry codes: (i) 
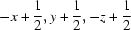
; (ii) 
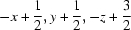
; (iii) 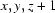.

**Fig. 1.**
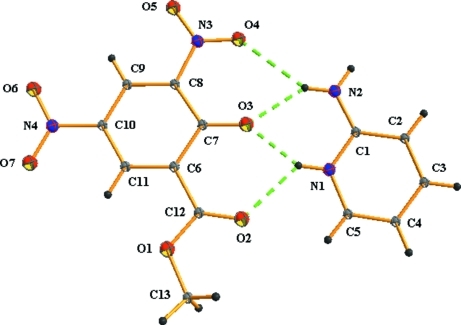
The molecular components of (I), showing the atom-labelling scheme. Displacement ellipsoids are drawn at the 50% probability level. Hydrogen bonds are shown as dashed lines

**Fig. 2.**
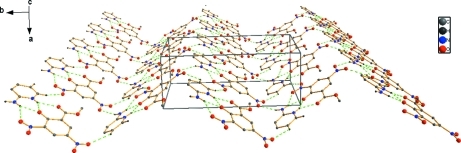
The extended 2D network of compound (I) formed by N-H···O and C-H···O hydrogen bonds

## supplementary crystallographic information

### Comment

The structures of many molecular proton transfer adducts containing substituted pyridinium and an acid anion have been reported in the past decades (Gellert & Hsu, 1988; Smith *et al.*, 2000; Jebas *et al.*, 2006; Rademeyer, 2007; Hemamalini & Fun, 2010*a*,*b*, *c*). As a substituted pyridinium, 2-aminopyridine has attracted great attention due to its various hydrogen bonds (Gellert & Hsu, 1988; Jebas *et al.*, 2006; Perpétuo & Janczak, 2010; Hemamalini & Fun, 2010*a*). We report here the synthesis and crystal structure of 2-aminopyridinium 3,5-dinitromethyl salicylate (I).

In the title compound, proton transfer has occurred from the hydroxyl group. As illustrated in Figure 1, the title molecule consists of a protonated 2-aminopyridinium cation and a 3,5-dinitromethyl salicylate anion. The cation and the anion are linked *via* two N—H···O(hydroxy), one N—H···O(carboxy) and one N—H···O(nitro group) hydrogen bonds to form an ion pair. The dihedral angle between the pyridinium ring in the cation and the benzene ring in the anion is 3.65 (6)°.

The bond lengths and angles in (I) are similar to those in other 2-aminopyridinium complexes (Jebas *et al.*, 2006; Perpétuo & Janczak, 2010; Hemamalini & Fun, 2010*a*).

As shown in Figure 2, the adduct at (*x*, *y*, *z*) connects to two neighboring adducts [at (0.5-*x*, 0.5+*y*, 0.5-*z*) and at (0.5-*x*, -0.5+*y*, 0.5-*z*)] through two N2-H···O6A (symmetry code A, 0.5-*x*, 0.5+*y*, 0.5-*z*) hydrogen bonds, forming a spiral chain. At the same time, the adduct at (*x*, *y*, *z*) interacts with two neighboring adducts *via* two C4-H···O7B (symmetry code B, 0.5-*x*, 0.5+*y*, 1.5-*z*) hydrogen bonds, also resulting in a spiral chain. A further C5-H···O5D (symmetry code D, *x*, *y*, 1+*z*) hydrogen bond connects the adduct to another two adducts. Therefore, every adduct connects to six neighboring adducts by these N-H···O and C_aryl_-H···O hydrogen bonds to yield an extended undulating two-dimensional network (Figure 2).

The benzene ring of the anion at (*x*, *y*, *z*) and the benzene ring in the anion at (1-*x*, 1-*y*, 1-*z*) are almost parallel, with a dihedral angle of 0.00 (6)° between them. The interplanar spacing is about 3.309 (1) Å, the centroid-centroid separation is 3.69 (1) Å, indicating a weak π-π interaction between these rings (Figure 3).

### Experimental

Reagents and solvents were used as obtained without further purification. 3,5-dinitromethyl salicylate was synthesized according to literature methods (Bartlett & Trachten, 1958). Ni(OAc)_2_^.^4H_2_O (0.0498g, 0.2 mmol) was dissolved in 10 mL of methanol to yield solution A. 3,5-dinitromethyl salicylate (0.0484 g, 0.2 mmol) and 2-aminopyridine (0.0188 g , 0.2 mmol) were dissolved in 10 mL of acetone to yield solution B. Solution A was slowly added to solution B. The mixture was stirred for 4 h at room temperature. After filtration, the green filtrate was allowed to stand at room temperature for several days. The yellow block crystals of the title compound (I) were obtained by slow evaporation.

### Refinement

The pyridinium H atom and H atoms in the NH_2_ group were located in a Fourier map and their positions refined. This resulted in the best placement of these atoms in the hydrogen-bonding network. All other H atoms were placed in calculated positions and refined using a riding model [C-H = 0.93 Å and Uiso(H) = 1.2Ueq(C) for aromatic H atoms, C-H = 0.96 Å and Uiso(H) = 1.5Ueq(C) for methyl H atoms].

### Crystal data

**Table:**

C_5_H_7_N_2_^+^·C_8_H_5_N_2_O_7_^−^	*F*(000) = 696
*M**_r_* = 336.27	*D*_x_ = 1.592 Mg m^−^^3^
Monoclinic, *P*2_1_/*n*	Mo *K*α radiation, λ = 0.71073 Å
Hall symbol: -P 2yn	Cell parameters from 3200 reflections
*a* = 7.4088 (3) Å	θ = 3.2–27.5°
*b* = 19.1779 (6) Å	µ = 0.13 mm^−^^1^
*c* = 9.9784 (4) Å	*T* = 293 K
β = 98.2825 (15)°	Block, pale yellow
*V* = 1403.00 (9) Å^3^	0.35 × 0.35 × 0.26 mm
*Z* = 4	

### Data collection

**Table:**

Rigaku R-AXIS SPIDER IP diffractometer	3200 independent reflections
Radiation source: fine-focus sealed tube	2760 reflections with *I* > 2σ(*I*)
graphite	*R*_int_ = 0.024
φ and ω scan	θ_max_ = 27.5°, θ_min_ = 3.2°
Absorption correction: ψ scan (*TEXRAY*; Molecular Structure Corporation, 1999)	*h* = −9→9
*T*_min_ = 0.951, *T*_max_ = 0.969	*k* = −24→24
21789 measured reflections	*l* = −12→12

### Refinement

**Table:**

Refinement on *F*^2^	Primary atom site location: structure-invariant direct methods
Least-squares matrix: full	Secondary atom site location: difference Fourier map
*R*[*F*^2^ > 2σ(*F*^2^)] = 0.040	Hydrogen site location: inferred from neighbouring sites
*wR*(*F*^2^) = 0.111	H-atom parameters constrained
*S* = 1.09	*w* = 1/[σ^2^(*F*_o_^2^) + (0.0608*P*)^2^ + 0.3452*P*] where *P* = (*F*_o_^2^ + 2*F*_c_^2^)/3
3200 reflections	(Δ/σ)_max_ = 0.001
221 parameters	Δρ_max_ = 0.32 e Å^−^^3^
0 restraints	Δρ_min_ = −0.30 e Å^−^^3^

### Special details

**Table:**

Geometry. All esds (except the esd in the dihedral angle between two l.s. planes) are estimated using the full covariance matrix. The cell esds are taken into account individually in the estimation of esds in distances, angles and torsion angles; correlations between esds in cell parameters are only used when they are defined by crystal symmetry. An approximate (isotropic) treatment of cell esds is used for estimating esds involving l.s. planes.
Refinement. Refinement of F^2^ against ALL reflections. The weighted R-factor wR and goodness of fit S are based on F^2^, conventional R-factors R are based on F, with F set to zero for negative F^2^. The threshold expression of F^2^ > 2sigma(F^2^) is used only for calculating R-factors(gt) etc. and is not relevant to the choice of reflections for refinement. R-factors based on F^2^ are statistically about twice as large as those based on F, and R- factors based on ALL data will be even larger.

### Fractional atomic coordinates and isotropic or equivalent isotropic displacement parameters (Å^2^)

**Table:**

	*x*	*y*	*z*	*U*_iso_*/*U*_eq_	
C1	−0.02842 (16)	0.69774 (6)	0.60013 (13)	0.0241 (3)	
C2	−0.11062 (17)	0.75222 (6)	0.66522 (14)	0.0289 (3)	
H2A	−0.1639	0.7897	0.6151	0.035*	
C3	−0.11154 (18)	0.74967 (7)	0.80192 (14)	0.0311 (3)	
H3A	−0.1657	0.7856	0.8445	0.037*	
C4	−0.03148 (18)	0.69327 (7)	0.87924 (13)	0.0307 (3)	
H4A	−0.0299	0.6919	0.9726	0.037*	
C5	0.04326 (17)	0.64096 (7)	0.81366 (12)	0.0267 (3)	
H5A	0.0952	0.6029	0.8625	0.032*	
C6	0.29417 (15)	0.45835 (6)	0.52321 (11)	0.0210 (2)	
C7	0.22285 (16)	0.51541 (6)	0.43454 (12)	0.0222 (2)	
C8	0.25155 (16)	0.50645 (6)	0.29470 (12)	0.0228 (2)	
C9	0.34193 (16)	0.45058 (6)	0.24919 (11)	0.0232 (2)	
H9A	0.3571	0.4473	0.1585	0.028*	
C10	0.41023 (15)	0.39918 (6)	0.34032 (12)	0.0215 (2)	
C11	0.38567 (16)	0.40290 (6)	0.47609 (12)	0.0214 (2)	
H11A	0.4314	0.3676	0.5355	0.026*	
C12	0.27237 (16)	0.46104 (6)	0.66889 (12)	0.0237 (2)	
C13	0.3255 (2)	0.40100 (9)	0.87787 (14)	0.0451 (4)	
H13A	0.3399	0.3538	0.9098	0.068*	
H13B	0.2093	0.4185	0.8942	0.068*	
H13C	0.4209	0.4294	0.9250	0.068*	
N1	0.04300 (14)	0.64367 (5)	0.67753 (10)	0.0233 (2)	
H01A	0.0871	0.6072	0.6366	0.044 (5)*	
N2	−0.01983 (16)	0.69663 (6)	0.46755 (11)	0.0301 (3)	
H02A	0.0212	0.6592	0.4298	0.043 (5)*	
H02B	−0.0560	0.7330	0.4189	0.043 (5)*	
N3	0.18614 (16)	0.55870 (6)	0.19326 (11)	0.0312 (3)	
N4	0.50832 (14)	0.34132 (5)	0.29295 (10)	0.0241 (2)	
O1	0.33525 (16)	0.40289 (5)	0.73434 (9)	0.0380 (3)	
O2	0.20907 (15)	0.50859 (5)	0.72572 (9)	0.0361 (2)	
O3	0.14378 (14)	0.56716 (5)	0.47530 (9)	0.0347 (2)	
O4	0.12739 (17)	0.61428 (5)	0.22518 (11)	0.0425 (3)	
O5	0.1928 (3)	0.54493 (9)	0.07588 (12)	0.1015 (8)	
O6	0.52421 (14)	0.33865 (5)	0.17125 (9)	0.0329 (2)	
O7	0.57286 (14)	0.29666 (5)	0.37464 (10)	0.0342 (2)	

### Atomic displacement parameters (Å^2^)

**Table:**

	*U*^11^	*U*^22^	*U*^33^	*U*^12^	*U*^13^	*U*^23^
C1	0.0235 (5)	0.0210 (5)	0.0277 (6)	−0.0042 (4)	0.0030 (5)	0.0003 (4)
C2	0.0288 (6)	0.0210 (6)	0.0366 (7)	0.0003 (5)	0.0031 (5)	−0.0011 (5)
C3	0.0305 (6)	0.0267 (6)	0.0367 (7)	−0.0006 (5)	0.0073 (5)	−0.0103 (5)
C4	0.0331 (7)	0.0338 (7)	0.0255 (6)	−0.0029 (5)	0.0057 (5)	−0.0063 (5)
C5	0.0288 (6)	0.0273 (6)	0.0237 (6)	−0.0016 (5)	0.0028 (5)	−0.0001 (5)
C6	0.0221 (5)	0.0229 (6)	0.0181 (5)	−0.0014 (4)	0.0027 (4)	−0.0004 (4)
C7	0.0239 (5)	0.0221 (5)	0.0206 (5)	0.0005 (4)	0.0029 (4)	−0.0010 (4)
C8	0.0257 (6)	0.0229 (6)	0.0194 (5)	−0.0001 (4)	0.0014 (4)	0.0028 (4)
C9	0.0258 (6)	0.0264 (6)	0.0175 (5)	−0.0016 (4)	0.0034 (4)	−0.0008 (4)
C10	0.0226 (5)	0.0203 (5)	0.0219 (6)	−0.0007 (4)	0.0046 (4)	−0.0027 (4)
C11	0.0225 (5)	0.0210 (5)	0.0206 (5)	−0.0017 (4)	0.0028 (4)	0.0012 (4)
C12	0.0248 (6)	0.0258 (6)	0.0206 (5)	0.0007 (4)	0.0034 (4)	0.0004 (4)
C13	0.0604 (10)	0.0566 (10)	0.0200 (7)	0.0201 (8)	0.0117 (6)	0.0107 (6)
N1	0.0261 (5)	0.0209 (5)	0.0233 (5)	0.0003 (4)	0.0053 (4)	−0.0014 (4)
N2	0.0410 (6)	0.0236 (5)	0.0261 (5)	0.0033 (4)	0.0062 (5)	0.0037 (4)
N3	0.0399 (6)	0.0313 (6)	0.0224 (5)	0.0076 (5)	0.0046 (5)	0.0052 (4)
N4	0.0256 (5)	0.0230 (5)	0.0244 (5)	−0.0013 (4)	0.0061 (4)	−0.0022 (4)
O1	0.0586 (7)	0.0379 (5)	0.0194 (4)	0.0191 (5)	0.0117 (4)	0.0066 (4)
O2	0.0546 (6)	0.0321 (5)	0.0237 (4)	0.0117 (4)	0.0125 (4)	−0.0005 (4)
O3	0.0507 (6)	0.0296 (5)	0.0246 (5)	0.0156 (4)	0.0084 (4)	0.0011 (4)
O4	0.0678 (7)	0.0245 (5)	0.0355 (5)	0.0100 (5)	0.0088 (5)	0.0069 (4)
O5	0.1943 (19)	0.0906 (11)	0.0208 (6)	0.0938 (13)	0.0190 (8)	0.0151 (6)
O6	0.0432 (5)	0.0334 (5)	0.0237 (4)	0.0050 (4)	0.0097 (4)	−0.0060 (4)
O7	0.0433 (5)	0.0269 (5)	0.0340 (5)	0.0102 (4)	0.0117 (4)	0.0055 (4)

### Geometric parameters (Å, °)

**Table:**

C1—N2	1.3338 (16)	C9—C10	1.3864 (16)
C1—N1	1.3543 (16)	C9—H9A	0.9300
C1—C2	1.4136 (17)	C10—C11	1.3945 (15)
C2—C3	1.3659 (19)	C10—N4	1.4429 (15)
C2—H2A	0.9300	C11—H11A	0.9300
C3—C4	1.409 (2)	C12—O2	1.2041 (15)
C3—H3A	0.9300	C12—O1	1.3414 (15)
C4—C5	1.3586 (18)	C13—O1	1.4448 (15)
C4—H4A	0.9300	C13—H13A	0.9600
C5—N1	1.3591 (15)	C13—H13B	0.9600
C5—H5A	0.9300	C13—H13C	0.9600
C6—C11	1.3796 (16)	N1—H01A	0.8952
C6—C7	1.4578 (16)	N2—H02A	0.8838
C6—C12	1.4864 (15)	N2—H02B	0.8702
C7—O3	1.2498 (14)	N3—O5	1.2087 (16)
C7—C8	1.4516 (16)	N3—O4	1.2112 (15)
C8—C9	1.3748 (17)	N4—O7	1.2307 (14)
C8—N3	1.4568 (15)	N4—O6	1.2378 (13)
			
N2—C1—N1	118.87 (11)	C9—C10—N4	119.04 (10)
N2—C1—C2	123.56 (11)	C11—C10—N4	119.99 (10)
N1—C1—C2	117.56 (11)	C6—C11—C10	120.65 (10)
C3—C2—C1	119.79 (12)	C6—C11—H11A	119.7
C3—C2—H2A	120.1	C10—C11—H11A	119.7
C1—C2—H2A	120.1	O2—C12—O1	122.13 (11)
C2—C3—C4	120.81 (12)	O2—C12—C6	126.26 (11)
C2—C3—H3A	119.6	O1—C12—C6	111.61 (10)
C4—C3—H3A	119.6	O1—C13—H13A	109.5
C5—C4—C3	118.15 (12)	O1—C13—H13B	109.5
C5—C4—H4A	120.9	H13A—C13—H13B	109.5
C3—C4—H4A	120.9	O1—C13—H13C	109.5
C4—C5—N1	120.74 (12)	H13A—C13—H13C	109.5
C4—C5—H5A	119.6	H13B—C13—H13C	109.5
N1—C5—H5A	119.6	C1—N1—C5	122.92 (10)
C11—C6—C7	121.65 (10)	C1—N1—H01A	118.4
C11—C6—C12	119.21 (10)	C5—N1—H01A	118.6
C7—C6—C12	119.11 (10)	C1—N2—H02A	120.2
O3—C7—C8	123.18 (11)	C1—N2—H02B	119.0
O3—C7—C6	122.99 (10)	H02A—N2—H02B	120.7
C8—C7—C6	113.83 (10)	O5—N3—O4	120.90 (12)
C9—C8—C7	123.75 (10)	O5—N3—C8	117.84 (11)
C9—C8—N3	115.83 (10)	O4—N3—C8	121.25 (11)
C7—C8—N3	120.41 (10)	O7—N4—O6	122.57 (10)
C8—C9—C10	119.11 (10)	O7—N4—C10	118.96 (10)
C8—C9—H9A	120.4	O6—N4—C10	118.47 (10)
C10—C9—H9A	120.4	C12—O1—C13	116.11 (10)
C9—C10—C11	120.97 (10)		
			
N2—C1—C2—C3	−179.20 (12)	C9—C10—C11—C6	−0.67 (17)
N1—C1—C2—C3	1.62 (18)	N4—C10—C11—C6	179.18 (10)
C1—C2—C3—C4	−0.03 (19)	C11—C6—C12—O2	173.68 (12)
C2—C3—C4—C5	−1.28 (19)	C7—C6—C12—O2	−4.57 (18)
C3—C4—C5—N1	0.98 (19)	C11—C6—C12—O1	−5.50 (16)
C11—C6—C7—O3	−178.03 (11)	C7—C6—C12—O1	176.25 (10)
C12—C6—C7—O3	0.17 (18)	N2—C1—N1—C5	178.80 (11)
C11—C6—C7—C8	2.18 (16)	C2—C1—N1—C5	−1.97 (17)
C12—C6—C7—C8	−179.61 (10)	C4—C5—N1—C1	0.68 (18)
O3—C7—C8—C9	178.46 (12)	C9—C8—N3—O5	10.0 (2)
C6—C7—C8—C9	−1.76 (17)	C7—C8—N3—O5	−170.96 (17)
O3—C7—C8—N3	−0.49 (18)	C9—C8—N3—O4	−169.76 (12)
C6—C7—C8—N3	179.30 (10)	C7—C8—N3—O4	9.27 (19)
C7—C8—C9—C10	0.18 (18)	C9—C10—N4—O7	178.17 (11)
N3—C8—C9—C10	179.17 (10)	C11—C10—N4—O7	−1.68 (16)
C8—C9—C10—C11	1.12 (17)	C9—C10—N4—O6	−1.80 (16)
C8—C9—C10—N4	−178.74 (10)	C11—C10—N4—O6	178.35 (10)
C7—C6—C11—C10	−1.09 (17)	O2—C12—O1—C13	−1.0 (2)
C12—C6—C11—C10	−179.29 (10)	C6—C12—O1—C13	178.21 (12)

### Hydrogen-bond geometry (Å, °)

**Table:**

*D*—H···*A*	*D*—H	H···*A*	*D*···*A*	*D*—H···*A*
N1—H01A···O3	0.90	1.88	2.6864 (13)	148.
N1—H01A···O2	0.90	2.23	2.8783 (14)	130.
N2—H02A···O3	0.88	2.01	2.7592 (14)	142.
N2—H02A···O4	0.88	2.45	3.2082 (15)	144.
N2—H02B···O6^i^	0.87	2.24	3.0537 (14)	155.
C4—H4A···O7^ii^	0.93	2.57	3.2052 (16)	126.
C5—H5A···O5^iii^	0.93	2.42	3.2604 (17)	151.

Symmetry codes: (i) −*x*+1/2, *y*+1/2, −*z*+1/2; (ii) −*x*+1/2, *y*+1/2, −*z*+3/2; (iii) *x*, *y*, *z*+1.
